# Maternal Mind-Mindedness Provides a Buffer for Pre-Adolescents at Risk for Disruptive Behavior

**DOI:** 10.1007/s10802-016-0165-5

**Published:** 2016-05-14

**Authors:** Claire Hughes, Amanda Aldercotte, Sarah Foley

**Affiliations:** Centre for Family Research, University of Cambridge, Cambridge, CB2 3RQ UK

**Keywords:** Mind-mindedness, Disruptive behavior, Adolescence, Five-minute speech sample

## Abstract

Maternal mind-mindedness, defined as the propensity to view one’s child as an agent with independent thoughts and feelings, mitigates the impact of low maternal education on conduct problems in young children (Meins et al. 2013), but has been little studied beyond the preschool years. Addressing this gap, we applied a multi-measure and multi-informant approach to assess family adversity and disruptive behavior at age 12 for a socially diverse sample of 116 children for whom ratings of disruptive behavior at age 6 were available. Each mother was asked to describe her child and transcripts of these five-minute speech samples were coded for (i) mind-mindedness (defined by the proportion of child attributes that were mental rather than physical or behavioral) and (ii) positivity (defined by the proportion of child attributes that were positive rather than neutral or negative). Our regression results showed that, independent of associations with prior adjustment, family adversity, child gender and low maternal monitoring, mothers’ mind-mindedness (but not positivity) predicted unique variance in disruptive behavior at age 12. In addition, a trend interaction term provided partial support for the hypothesis that pre-adolescents exposed to family adversity may benefit in particular from maternal mind-mindedness. We discuss the possible mechanisms underpinning these findings and their implications for clinical interventions to reduce disruptive behavior in adolescence.

Mind-mindedness, defined as the propensity to view others as mental agents with their own thoughts, feelings and desires (e.g., Meins et al. 2003), is a concept that has attracted increasing attention within research into the impact of parental sensitivity on child outcomes. Although early attachment research defined parental sensitivity as encompassing an awareness of the child’s point of view (Ainsworth et al. 1974), subsequent studies (e.g., Belsky 1984) focused on behavioral markers of parental sensitivity (e.g., prompt, appropriate and consistent responses to the child’s needs). However, findings from several independent studies highlight the value of examining parents’ sensitivity to children’s psychological rather than physical needs (Meins et al. 2001).

Within this field, there is an interesting debate regarding how the construct of mind-mindedness should be understood. In particular, is mind-mindedness a trait, which may be related to other maternal characteristics (e.g., education, wellbeing) or is it instead an index of relationship quality, related to other relationship markers (e.g., closeness)? To address this question, Meins et al. (2014) designed an innovative set of studies in which participants provided verbal or written descriptions of: (1) their child; (2) a close friend and current romantic partner; (3) two famous people, two works of art and a close friend; (4) two famous people (specified and own choice) and a close friend. They found that descriptions of celebrities and works of art generated fewer mind-minded descriptions than those of individuals with whom the participant had a close relationship. Of particular interest is the contrasting relationships between individual’s descriptions. Specifically, participants’ tendency to invoke mind-related descriptions of their close friends was positively correlated with mind-minded descriptions of romantic partners but not with the frequency of mind-minded descriptions of either famous individuals or works of art. The authors concluded that maternal mind-mindedness is not a trait but rather a facet of close relationships. In support of this view several independent studies have reported no association between mind-mindedness and maternal education (e.g., Bernier et al. 2010).

However, as Meins et al. (2014) acknowledge, other studies have reported a significant association between mind-mindedness and maternal education and socio-economic status (e.g., Bordeleau et al. 2012; Lundy 2013). A study by Reznick (1999) also indicates interesting cultural contrasts. In this study, 130 mothers of 9–10 month old infants watched 28 short video clips of babies and rated the intentionality of the babies’ actions. Alongside contrasts that related to the characteristics of the babies in the video clips (older and female babies were perceived as more intentional), this study also showed that Hispanic-American mothers gave lower ratings of intentionality than either European-American or African American mothers. It therefore remains possible that maternal characteristics at least partially underpin variation in mind-mindedness.

## Mind-Mindedness and Disruptive Behavior

Of particular relevance to the current study is a recent longitudinal investigation conducted by Meins et al. (2013) that tracked 171 mother-infant dyads from 8- to 61-months of age. Observational ratings of maternal mind-mindedness at the first time-point were examined as predictors of child externalising problems at age 61 months (as rated by mothers). Their findings showed that this predicted association was only evident for the 60 families in the two groups with the lowest socio-economic status (SES; i.e., parents with no post-16 education and either unemployed or in unskilled/semi-skilled employment). Echoing this, Brophy-Herb et al. (2015) also found mental state talk to be most beneficial for toddlers from high-risk families. Specifically, toddlers’ disruptive behaviour reduced over time if their mothers’ used high levels of emotion talk during a book-sharing task but only if they were from high-risk backgrounds. Whilst adopting two different methods, both research groups conclude the use of mind-related talk mitigates the impact of low SES on young children’s conduct problems.

The first two aims of the current study were to test whether, in this sample of 12-year-olds: (i) disruptive behavior would show a unique association with low levels of maternal mind-mindedness; and (ii) whether this association would be particularly salient in children at elevated risk for disruptive behavior. For each of these two aims (described in more detail below), our goal was to extend the developmental scope of existing research with pre-schoolers.

## Does Parental Mind-Mindedness Matter for Older Children?

Numerous studies have assessed mind-mindedness in parents (typically mothers) of infants (Bernier et al. 2010; Meins et al. 2001) and a few studies (Meins et al. 2014; Walker et al. 2012) have examined mind-mindedness in parents of pre-schoolers or early school-aged children. To our knowledge, no study has yet examined parental mind-mindedness beyond middle childhood. This restricted developmental focus is significant for both practical and theoretical reasons. For parents faced with crying infants, toddler tantrums or pre-schoolers’ defiance, an obvious challenge is to get inside the head of their child; thus it is easy to see that variation in parental mind-mindedness may be crucial in explaining individual differences in child adjustment in the early years. Whether parental mind-mindedness still matters as children enter early adolescence is, however, an open question.

On the one hand, children make remarkable gains in their ability to communicate their thoughts and feelings across the first decade of life (Apperly et al. 2009; Hughes 2011) such that, while infant cues can be easily misunderstood, the task of tuning into children’s inner world becomes much less taxing as they grow up. In addition, within all relationships, adults’ views of their social partners are quick to form and difficult to change (e.g., Sunnafrank and Ramirez 2004). Thus the association between parents’ propensity to view their children as mental agents and children’s current adjustment may well become non-significant once prior levels of adjustment are taken into account. For these two reasons at least, the links between parental mind-mindedness and adjustment may be restricted to early childhood.

The competing hypothesis is that parental mind-mindedness is equally salient in adolescence. According to Darling and Steinberg’s (1993) integrative model of parenting, the emotional climate of the parent–child relationship (which depends upon parental sensitivity) provides an overarching context that moderates the effects of parenting practices. Thus if mind-mindedness is a key component of parental sensitivity, it is likely to be fundamentally important for child adjustment and therefore might be predicted to show an enduring influence on child outcomes. This is the premise of the current study.

One might even make a stronger argument for the importance of mind-mindedness in parents of young adolescents. In particular, theorists have long argued that the key developmental task for adolescence is one of identity formation (Erikson 1968). While pre-schoolers and primary-school aged children are typically willing to align themselves with their parents’ views of the world, by early adolescence they become keen to distinguish their own thoughts and feelings from those of others. Thus early adolescence may well be a period in which parents’ ability and willingness to see their children’s inner worlds is particularly important for relationship quality and child adjustment. At a theoretical level, this hypothesis is supported by self-determination theory, in which the growth of autonomy, a key developmental task for young adolescents, is not simply a matter of increased independence or emotional and practical separation from adult caregivers, but rather the development of clear interests and values (Ryan et al. 1995). This development is best fostered by parents who are empathic to their children’s perspective and help their children explore and act on their true personal values and interests (Soenens et al. 2007). Reflecting this importance, researchers have shown that parenting that is characterized by an unwillingness to grant children agency and autonomy (i.e., high in psychological control) predicts both depression and conduct problems in early adolescence (Aunola and Nurmi 2005). Indeed, meta-analytic findings indicate that parental psychological control ranks alongside poor parental monitoring and parental rejection or hostility as the strongest predictor of delinquency in early adolescence (Hoeve et al. 2009).

## Is Parental Mind-Mindedness Especially Important for Children at Risk?

A vast body of research demonstrates that risk factors typically display a cumulative impact on child outcomes (e.g., Loeber and Hay 1997; Morales and Guerra 2006), such that any single factor is only likely to explain part of the variation in adjustment in early adolescence (Jaffee et al. 2007). While Meins et al. (2013) report of the role of mind-mindedness in reducing the association between low SES and preschoolers’ disruptive behavior is intriguing, further work is needed to establish whether this buffering effect is evident in older children and also significant with regards to a broader set of risk markers. Thus, in the current study we controlled for teachers’ ratings of disruptive behaviour at age 6, as well as child gender and low parental monitoring, and examined mind-mindedness in relation to family adversity (as indexed by financial strain, poor housing, solo parenthood, low maternal education, and maternal depressive symptoms). This enabled us test the independence and specificity of the association between low maternal mind-mindedness and children’s disruptive behavior at age 12.

At this point it is worth noting that parental mentalization about children can be measured in a number of different ways (see Schiborr et al. 2013). One of the earliest methods adopted involved coding mother-infant interactions for appropriate references to the infants’ goals or mental states. However, childhood problems of disruptive behavior are known to have a powerful influence on the quality of parents’ interactions with their children (Alemany et al. 2013). As a result, ‘off-line’ ratings based on how parents talk *about* rather than *to* their child may be more useful. In this study we therefore elected to follow an alternative representational approach in which mind-mindedness is coded from parents’ open-ended descriptions of their child (Meins and Fernyhough 2015). To maximise variability, we employed the five-minute speech sample (FMSS), a clinical method that has been widely adopted in the developmental literature (for a review, see Sher-Censor 2015).

Parents vary dramatically in how easy or difficult they find the task of talking about their child for five minutes and so, in order to control for variation in parental fluency we coded not only the number of mental comments but also the number of non-mental comments, using the ratio between these as our index of parental mind-mindedness. Recently, Demers et al. (2010) have argued that it is not mind-mindedness per se that matters for child adjustment, but rather positive mind-mindedness – that is, the ability to perform the dual task of adopting a positive stance *and* attending to children’s mental states. A further goal of the current study was therefore to adopt directly comparable measures of maternal mind-mindedness and maternal positivity in order to examine them in tandem as predictors of adjustment in early adolescence.

In sum, the present study had two main aims. Our first aim was to extend the developmental scope of existing research by examining whether variation in maternal mind-mindedness, defined as the ratio of mental to non-mental child attributes given by mothers within the FMSS, predicted variance in children’s disruptive behavior in early adolescence and if so, whether this association remained significant when we controlled for effects of (i) prior adjustment, child gender, and low maternal monitoring; (ii) social adversity (as indexed by financial strain, poor housing, solo parenthood, low maternal education, and maternal depressive symptoms); and (iii) variation in a parallel measure of maternal positivity. Our second aim was to investigate the interplay between maternal mind-mindedness and family adversity as predictors of poor adjustment.

## Methods

### Participants

The sample for this study consisted of 116 children (55 % male) in Year 7 (i.e., first year of secondary school) for whom: a) teachers had provided ratings of disruptive behavior when children were in Year 1 of primary school and were 6-years-old, *M* = 6.03, *SD* = 0.35; and b) mothers had completed the FMSS that was coded for mind-mindedness at age 12, *M* = 12.16, *SD* = 0.29. Fourteen children had received a clinically relevant diagnosis of Conduct Disorder, Autism Spectrum Disorder or Attention-Deficit-Hyperactivity Disorder (*n* = 2, *n* = 10, *n* = 2, respectively). Reflecting the local population, the study sample was predominantly White (just 3.4 % children had at least one parent from an ethnic minority) but diverse in terms of socio-economic status. In particular, only 43 % of the mothers had post-18 education. Ethical approval was granted from our University Psychology Research Ethics Committee and written informed consent was obtained from each participant.

### Procedure

Multi-informant data for this study were gathered at two study time-points, separated by an interval of 6 school years. Time-1 ratings of disruptive behavior were gathered from teachers when the children were around age 6. Time-2 measures were gathered during face-to-face interviews conducted with the mothers when the study children were around age 12. At this time-point, two researchers visited the individual families in their home.

### Measures

#### Control Measures

Three important control variables were considered in the current study, the first two pertained to child factors while the third reflected the quality of maternal monitoring. The first child-related factor that we included in our model was child gender, as a number of studies have demonstrated gender differences in disruptive behaviour (Maughan et al. 2004). The second child-related factor acknowledged the developmental stability of problem behavior. Specifically, at age 6 (i.e., approximately 6 years before the home visits conducted for the current study) teachers rated children’s conduct problems and hyperactivity using the five-item subscales of the Strengths and Difficulties Questionnaire (SDQ; Goodman 1997). Both subscales demonstrated adequate internal consistency, Cronbach’s α = 0.56 and 0.85, respectively, and were significantly correlated, *r* = 0.62, *p* < 0.001; thus, the mean scores on these two subscales were averaged to create an index of children’s previous problem behavior.

The final control variable included in the current study was mothers’ monitoring of their children’s activities and behavior. Maternal monitoring was included for two reasons, the first being its relevance to children’s behavioral adjustment as they enter the adolescent years (Dishion and McMahon 1998). The second reason for including maternal monitoring was to distinguish maternal mind-mindedness and positivity from this aspect of the parent-child relationship and mothers’ *parenting style* in general. At the age 12 visit, mother’s reported on their own levels of parental monitoring by rating ten behaviors on a five-point scale, for example how often the child goes out at night without a set home time, on the Alabama Parenting Questionnaire (APQ; Shelton et al. 1996), Cronbach’s α = 0.81. Higher scores on the APQ reflected lower levels of maternal monitoring.

#### Disruptive Behavior at Age 12

During the home visit we also gathered multi-measure multi-informant ratings of the young adolescents’ disruptive behavior from three different sources. First, mothers provided ratings of conduct problems and hyperactivity on the (SDQ; Goodman 1997, Cronbach’s α = 0.72 and 0.81 respectively). Second, the two researchers independently completed post-visit ratings of difficult child behavior (four items, each on a 0- to 2-point scale); for example, whether or not the child was aggressive towards a sibling or disrespectful towards their mother. These ratings were averaged across the two researchers, Cronbach’s α = 0.80. Third, the 12-year-old participants themselves provided information on their engagement in both bullying and disruptive behavior on two questionnaire subscales: (i) the six-item subscale of the Peer Relationships Questionnaire (Rigby and Slee 1993) which had a possible range of 0–24 points and showed good internal consistency, Cronbach’s α = 0.83; and (ii) the six-item Behavioral Competence subscale of the Harter Self-Perception Profile (Cronbach’s α = 0.88, Harter 1982).

#### Maternal Speech Sample

During the home visits, we asked each mother to speak about her child and their relationship for five minutes (FMSS - Magana et al. 1986). These were elicited using the following standard instructions: “I’d like to hear your thoughts and feeling about (child’s name), in your own words and without my interrupting with any questions or comments. When I ask you to begin I’d like you to speak for five minutes, telling me what kind of a person (child’s name) is and how the two of you get along together. After you begin, I prefer not to answer any questions until after the five minutes are over. Do you have any questions before we begin?” Each FMSS was then transcribed and coded (by the first and third authors of this paper). Excluding ‘filler talk’ (e.g., “this is hard, what else can I say?”) all comments were categorized as either child or self-focused. Child-focused comments were then categorized as either mental (i.e., including a reference to the child’s cognitive states, emotional states or desire states) or non-mental (including behavioral attributes, physical attributes and general attributes of the child). This coding followed the same scheme as that used by Meins and Fernyhough (2015). In addition, however, the comments within each of these categories were also coded by valence (positive, neutral, negative) (Demers et al. 2010). Simple repetitions were not included, but different expressions of the same construct (e.g., He’s very smart/ he’s much cleverer than me/ he’s really got a gift for academic work) were counted as separate comments. Inter-rater reliability was established by independent double coding of 28 transcripts (24 % of the total sample) and yielded adequate intra-class correlations (ICC) for both mental attributes, ICC = 0.92, and non-mental attributes, ICC = 0.73, and for positive attributes, ICC = 0.85, and neutral/negative attributes, ICC = 0.73.

From this coding we extracted two parallel measures, each expressed as a ratio to control for differences in maternal verbosity. Specifically, the ratio of mental to non-mental child attributes included in each FMSS was used to index *maternal mind-mindedness* while the ratio of positive to neutral/negative child attributes included in each FMSS was used to index *maternal positivity*.

#### Family Adversity

To reflect the diverse aspects of family life, our family adversity factor included five measures that were gathered during the age 12 home visit:

- *Child report*:

(i) The four-items of the Family Affluence Scale (Currie et al. 2008) provide information about the numbers of computers in the child’s home (0, 1, 2+), the numbers of cars currently owned (0, 1, 2+),the number of family holidays away from home in past year (0, 1, 2+) and whether the child has a bedroom of his/her own (no =1, yes =2). Responses to the items were summed and reversed.

- *Maternal report*:

(ii) The number of years each child had spent in a lone-parent household;
(iii) Maternal depressive symptoms, rated using the 21-item Beck Depression Inventory (Beck et al. 1961), which showed good internal consistency, Cronbach’s α = 0.92;
(iv) Age at which mothers left education (reversed).

- *Researcher report*:

(v) Using a three-point (*no*, *somewhat or yes*) post-visit rating scale, researchers answered five questions about the state of the home; for example, were the rooms clean and safe or overcrowded. Items were reversed as necessary, so that a high score indicated a poorly maintained home. Note that ratings for a further two items about the garden and the child’s bedroom were dropped because there was too much missing data. Raters showed good agreement on the other items and the scale showed good internal consistency, Cronbach’s α = 0.93.

## Results

### Analytic Strategy and Data Reduction

We used multiple linear regression to examine whether maternal mind-mindedness was an independent predictor of children’s disruptive behavior and to explore the interaction between maternal mind-mindedness and family adversity as predictors of children’s disruptive behavior. Given the number of assessments used, we opted to conserve statistical power by first examining the validity of our measures of family adversity and disruptive behavior in separate confirmatory factor analyses (CFA) before building our regression model. All analyses were conducted in *Mplus* version 7.1 (Muthén and Muthén 2012) utilizing full information maximum likelihood to account for missing data. The following indices were used to ascertain model fit (Brown 2006): Root Mean Square Error of Approximation (RMSEA) < 0.06, Comparitive Fit Index (CFI) > 0.90, Tucker Lewis Index (TLI) > 0.90, and Standardized Root Mean Square Residual (SRMR) criterion of < 0.08. Factor scores representing family adversity and disruptive behavior were extracted from the CFA models to be used in subsequent regression analyses.

Our first CFA including ratings of 12-year-olds’ disruptive behavior from mothers, researchers, and self-report measures demonstrated a very good fit to the data, RMSEA = 0.00, CFI = 1.00, TLI = 1.05, SRMR = 0.01. The individual measures of disruptive behavior at age 12 were well correlated (see Table 1) and all indicator loadings were greater than 0.600 and significant at the *p* < 0.001 level. A second CFA representing the five measures of family adversity (financial strain, poor housing, solo parenthood, low maternal education, and maternal depressive symptoms) showed a good fit to the data, RMSEA = 0.02, CFI = 0.993, TLI = 0.987, SRMR = 0.04. The majority of the indicators were significantly correlated (see Table 2) and all five indicators significantly contributed to the family adversity factor at the *p* < 0.05 level. Although the factor loading of solo parenthood was only 0.270 (i.e., less than the standard cut-off of 0.400), we retained this indicator because it was significantly related to maternal ratings of depression, *r* = 0.27, *p* < 0.01, and to maintain a robust description of the life in the family home.

**Table 1:** Correlations between individual measures of children’s previous problem behavior at age 6 and disruptive behavior age 12

	1.	2.	3.	4.	5.	6.	7.
1. Age 6 SDQ conduct problems ^T^							
2. Age 6 SDQ hyperactivity ^T^	0.62***						
3. Age 12 SDQ conduct problems ^M^	0.34***	0.25*					
4. Age 12 SDQ hyperactivity ^M^	0.44***	0.49***	0.59***				
5. Age 12 Post-visit ratings disruptive behavior ^R^	0.35***	0.27**	0.41***	0.44***			
6. Age 12 PRQ bullying ^C^	0.30**	0.32**	0.16	0.23*	0.37***		
7. Age 12 HSPP behavioral competence (rev) ^C^	0.37***	0.31**	0.54***	0.51***	0.40***	0.43***	
*M*	0.95	3.41	0.37	0.65	0.21	1.12	−2.85
*SD*	1.31	2.91	0.39	0.54	0.34	0.30	0.44

SDQ = Strengths and difficulties questionnaire; PRQ = Peer relationships questionnaire; HSPP = Harter self-perception profile; T = Teacher; M = Mother; C = Child; R = Researcher. (rev) Denotes scales that are reverse scored

*p < 0.05

**p < 0.01

***p < 0.001

#### Preliminary Analyses

Table 2 presents means (M) and standard deviations (SD) alongside zero-order correlations for control variables and the factor scores representing children’s disruptive behavior at age 12 and family adversity. Children’s disruptive behavior at age 12 was significantly related to child gender, *r* = −0.32, *p* < 0.001, prior adjustment at age 6, *r* = 0.48, *p* < 0.001, and low maternal monitoring, *r* = 0.38, *p* < 0.001. Specifically, factor scores for disruptive behavior were higher for boys: *M* = 0.08, *SD* = 0.30, than for girls, *M* = −0.11, *SD* = 0.21; *t* (117) = 3.83, *p* < 0.001. As such, all three of these control variables were included in subsequent analyses.

**Table 2:** Summary of means, standard deviations, and zero-order correlations for all study variables

	1.	2.	3.	4.	5.	6.	7.	8.	9.	10.	11.	12.
1. Child gender	−											
2. Age 6 problem behavior ^T^	−0.32**	−										
3. Low maternal monitoring ^M^	0.12	0.09	−									
4. Family adversity	−0.04	0.36**	0.14	−								
5. Family affluence scale (rev)^C^	0.03	0.08	0.13	0.61***	−							
6. (Poor) State of home ^R^	−0.01	0.10	0.00	0.63***	0.13	−						
7. Maternal education (rev) ^M^	−0.06	0.12	0.14	0.71***	0.39***	0.32**	−					
8. Maternal depression ^M^	0.02	0.22*	0.11	0.78***	0.32**	0.36***	0.32**	−				
9. Lone parent (years) ^M^	−0.05	0.26**	−0.09	0.34***	0.05	0.14	0.14	0.27**	−			
10. Maternal positivity ^R^	0.08	−0.20*	−0.14	−0.15	−0.06	0.04	−0.31**	−0.10	−0.07	−		
11. Maternal mind-mindedness ^R^	−0.04	0.08	−0.11	−0.27*	−0.14	−0.13	−0.29**	−0.18^+^	−0.05	0.08	−	
12. Age12 disruptive behavior ^M,C,R^	−0.33***	0.48***	0.38***	0.41***	0.21*	0.12	0.37***	0.30**	0.20*	−0.25**	−0.26**	−
*M*		2.18	1.70	0.00	2.72	2.42	10.44	7.86	2.23	4.64	0.78	0.00
*SD*		1.92	0.46	−0.01	1.83	4.27	3.42	8.44	3.88	7.83	0.44	−0.07

The individual measures of the family adversity composite are reported alongside the composite score due to the diversity of the factors these measures represent; however, individual measures assessing the same construct across different raters (i.e., disruptive behavior) are reported in Table 1. T = Teacher; M = Mother; C = Child; R = Researcher. (rev) Denotes scales that are reverse scored

^+^ *p* < 0.10

*p < 0.05

**p < 0.01

***p < 0.001

### Does Maternal Mind-Mindedness independently Predict Age 12 Disruptive Behavior?

**Fig. 1:**
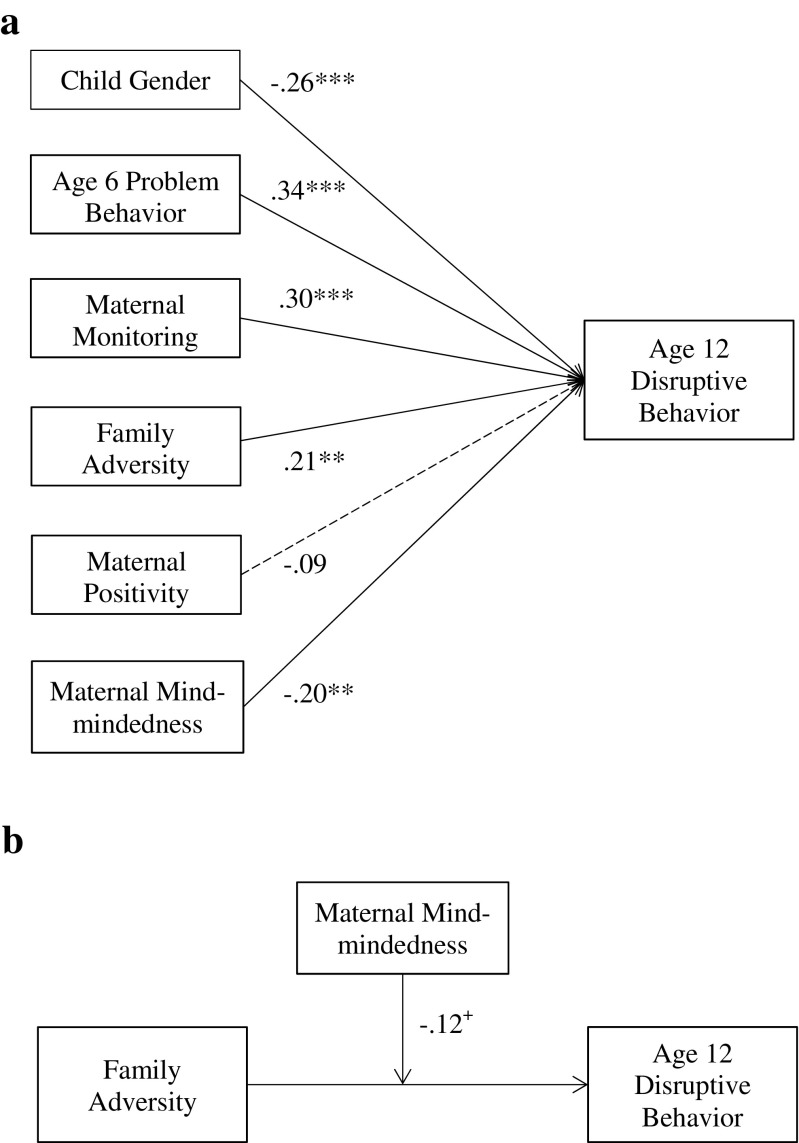
**a** Regression model depicting individual paths of maternal mind-mindedness and maternal positivity alongside control variables; **b** Model representing the strength of the interaction between family adversity and maternal mind-mindedness; ^+^ *p* < 0.10; **p < 0.01; ***p < 0.001

Having created factor scores representing age 12 disruptive behavior and family adversity and assessed the weight of our control variables (child gender, problem behavior at age 6, and low maternal monitoring), we built a multiple linear regression model to examine whether (i) maternal mind-mindedness independently predicts children’s disruptive behavior, and (ii) this association is separate from the contribution of maternal positivity. This model represented an adequate fit to the data, RMSEA = 0.05, CFI = 0.978, TLI = 0.965, SRMR = 0.05, and explained significant variance in children’s disruptive behavior, *R* ^2^ = 0.53, SE = 0.06, *z* = 8.31, *p* < 0.001. Moreover, except for maternal positivity all included pathways were significant (see Fig. 1a). As hypothesized, low maternal mind-mindedness predicted unique variance in ratings of children’s disruptive behavior at age 12, *β* = −0.20, SE = 0.07, *p* = 0.003. To confirm that the relation between mind-mindedness and disruptive behavior was distinct from the contribution of maternal positivity, we calculated the variance inflation factor (VIF) for maternal mind-mindedness, VIF = 1.12 (values greater than 10 indicate multi-collinearity - Kutner et al. 2004).

### Does Maternal Mind-Mindedness Buffer Children at Risk for Disruptive Behavior?

**Fig. 2:**
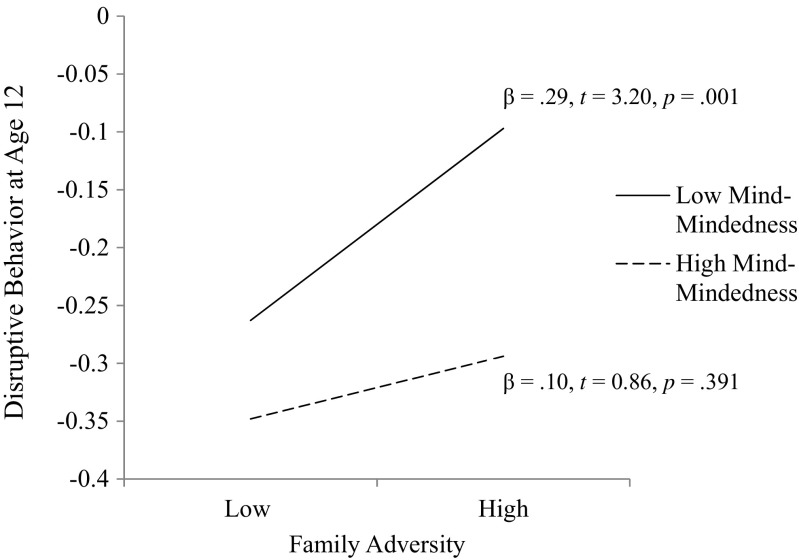
Visual representation of the trend interaction between maternal mind-mindedness and family adversity on age 12 disruptive behavior

Next we added an interaction term between family adversity and maternal mind-mindedness to the above regression model to examine the interplay between maternal mind-mindedness and family adversity in predicting children’s disruptive behavior. Both independent variables were mean-centered prior to calculating the interaction term. To maintain a parsimonious approach and conserve statistical power, maternal positivity was removed from this regression model. The model showed a good fit to the data, RMSEA = 0.00, CFI = 1.00, TLI = 1.01, SRMR = 0.04, and provided partial support for our second study hypothesis as the interaction term between family risk and maternal mind-mindedness was significant at a trend level, *β* = −0.12, SE = 0.07, *p* = 0.090 (see Fig. 1b). Post-hoc exploration of this trend followed the simple slope analysis procedure prescribed by Holmbeck (2002). In short, this procedure involves: (i) computing two new variables to represent low (−1SD) and high (+1SD) values of the family adversity moderator variable; (ii) calculating the interaction terms associated with each of these conditions; and (iii) substituting these new variables into separate regression models to calculate the slope of each condition. In this approach, the beta-values associated with the maternal mind-mindedness variable indicate whether the slopes of these conditional models are significant (see Fig. 2). While both models showed a good fit to the data, low maternal family adversity RMSEA = 0.00, CFI = 1.00, TLI = 1.01, SRMR = 0.04; high family adversity RMSEA = 0.00, CFI = 1.00, TLI = 1.01, SRMR = 0.06, maternal mind-mindedness was only significant in the high family adversity model, *β* = −0.37, SE = 0.12, *p* = 0.003. This result clarifies the trend moderation described above: the negative association between disruptive behavior and maternal mind-mindedness was stronger in the context of family adversity.

## Discussion

### Summary of Results

The current study of 116 children followed from age 6 to age 12 included three sets of measures, which were used to assess: (i) *disruptive behavior at age 12*, indexed by a multi-measure, multi-informant (research, parent, child) ratings and verified using confirmatory factor analysis; (ii) *family adversity*, indexed by a factor score for adversity that combined a variety of indicators (financial strain, poor housing, solo parenthood, low maternal education, and maternal depressive symptoms); and (iii) *maternal mind-mindedness and positivity*, coded from transcripts of mothers’ 5-min speech samples. Our analyses yielded two main findings. First, over and above the significant effect of child predictors (male gender and age-6 disruptive behavior), low maternal monitoring, family adversity, and maternal positivity, individual differences in maternal mind-mindedness predicted unique variance in disruptive behavior at age 12. Second, there was a marginally significant interaction that indicated that it was only in the context of family adversity that maternal mindedness predicted reduced disruptive behavior. Below, we relate these findings to previous work and discuss possible mechanisms underpinning each effect.

### How might Maternal Mind-Mindedness Help Reduce Disruptive Behavior in Pre-Adolescence?

Previous work on the interplay between parental attitudes and practice has focused heavily on families with young children (Kochanska 1990). The current findings therefore extend both the developmental and conceptual scope of the field by demonstrating a robust association between variation in parental representations of their children as agents with thoughts, feelings and desires and variation in behavioral adjustment in pre-adolescence. Moreover, whilst previous studies have reported robust links between child disruptive behavior and both maternal education and maternal depression (Ensor et al. 2012; Hughes and Ensor 2005), the current finding suggests an independent association with mind-mindedness. That is, although child disruptive behavior at age 12 showed significant associations with four other maternal measures (education, depression, low monitoring of child activities and positivity in the speech sample), child gender, and previous disruptive behaviour, mind-mindedness remained a significant predictor of child disruptive behavior when these predictors were all entered together. What mechanisms might underpin the association between maternal mind-mindedness and child adjustment? At least three possibilities deserve mention.

First, mothers who are more in tune with their children’s inner states may be better able to both pre-empt and defuse everyday conflict situations. This simple proposal has good face validity: as most parents know, maintaining harmony at home often rests on recognizing the signs of internal states, such as hunger, anxiety, loneliness or tiredness, which constrain the child’s ability to remember and comply with family rules. Second, variation in maternal mind-mindedness may facilitate developments in children’s self-concepts and reflective self-awareness, which in turn may contribute to their behavioral adjustment. Empirical support for this proposal comes both from studies of the intergenerational transmission of anxiety (for a review, see Bögels and Brechman-Toussaint 2006) and from the finding that prosocial adolescents showed self-concepts that were particularly likely to incorporate parental images of themselves (Hart and Fegley 1995). Third, children with conduct problems are known to perform poorly on tests of theory of mind (Hughes et al. 2000; Sharp and Venta 2013), such that another more developmental account hinges upon the importance of family talk about mental states for children’s growing understanding of mind. In previous work involving a subset of the current study sample (Ensor et al. 2013) it was shown that individual differences in the frequencies of mothers’ conversational references to mental states are stable across a four year period from toddlerhood to early school age and predict children’s later understanding of mind, even across the 8-year interval from age 2 to age 10 (Hughes and Devine 2014).

It therefore seems reasonable to suppose that mothers who often refer to mental states when talking *about* their child also frequently refer to mental states when talking *with* their child, such that ‘on-line’ mind-mindedness may mediate the association between low mind-mindedness as rated from mothers’ descriptions of their child and elevated disruptive behavior. Indeed, a similar mediation effect has been reported for pre-schoolers, with theory of mind as the outcome rather than disruptive behavior (Lundy 2013). Taken together, these findings suggest a possible cascade effect, in which maternal mind-mindedness influences family talk about people’s thoughts and feelings, which in turn enables children to develop the understanding of others needed to negotiate challenging social situations without resorting to displays of aggressive or negative behavior.

### Strengths of the Study

The main strength of the current study lies in its extension of the developmental scope of existing work. That is, while Meins et al. (2013) have reported a link between low maternal mind-mindedness and pre-schoolers’ disruptive behavior, the current study indicates that this relationship also holds true in pre-adolescence. Second, the longitudinal design of this study enabled us to control for prior levels of disruptive behavior. The across-time association in our multi-informant aggregate index of disruptive behavior was remarkably strong.

A further strength of the current study was its use of multi-informant measures. In particular, the focus on pre-adolescents rather than pre-schoolers enabled us to include children’s own self-reports in our measure of disruptive behavior. Including this control variable alongside gender was useful, as our findings indicated that both measures were equally strongly related to individual differences in disruptive behavior.

Demonstrating an effect of maternal mind-mindedness that was independent of child risk factors, family adversity and low maternal monitoring underscores the significance of maternal mind-mindedness. In addition, by exploring the interaction between maternal mind-mindedness and family adversity as predictors of disruptive behavior we were able to demonstrate a marginal protective effect, such that the association between mind-mindedness and disruptive behavior was particularly strong in the context of family adversity.

### Limitations of the Study

At least two limitations of the current study deserve note. First, although we were able to construct a multi-measure, multi-informant aggregate index of disruptive behavior our informants (child/mother/researcher) did not include teachers and did not explicitly focus on behavior outside the home. As a result, our ratings are likely to hinge upon the frequency and severity of the study children’s involvement in everyday conflict at home, rather than more generalized problems of externalizing behavior in other settings. Second, practical constraints, notably the timing of the home visits to coincide with children’s return from school, meant that we were unable to assess mind-mindedness in *fathers*. This is unfortunate as fathers may play a particularly interesting role in influencing the content and form of family talk (Bhavnagri and Parke 1991).

Interestingly, the two studies in this field to include fathers as well as mothers have produced somewhat contrasting findings. Using the construct of parental reflective functioning that, unlike mind-mindedness also includes parents’ reflections on their own experiences, thoughts and feelings, Benbassat and Priel (2012) found that it was only in this context of high parental reflective function that maternal and paternal warmth were associated with more positive adolescent social self-concepts. Likewise, adolescents’ negative self-concepts and externalizing problems were associated with paternal control (i.e., controlling, intrusive, and overprotective parenting), but only in the context of low paternal reflective function. In contrast, in a study of preschoolers, Lundy (2013) reported that mind-minded interactions mediated the association between children’s theory-of-mind performance and off-line mind-mindedness in mothers but not fathers. These diverging findings from studies of different age groups underscore the importance of adopting a developmental perspective when examining links between paternal and maternal cognitions on child outcomes.

### Future Directions

In considering the findings reported by Benbassat and Priel (2012) and Lundy (2013) it is worth noting that these two studies adopted different methods of assessing parental cognitions, focusing on reflective functioning and mind-mindedness, respectively. Very recently, Barreto et al. (2015) have documented contrasts in the correlates of parental mentalizing (assessed via a visual joke task) and mind-mindedness (assessed via a ‘describe your child’ task) and concluded that these are distinct constructs (see also Meins et al. 2014). To our knowledge, reflective function and mind-mindedness have yet to be assessed in a single study; this is needed in order to elucidate their relative overlap and independence as predictors of child outcomes. In the current study, for example, the mind-mindedness coding did not distinguish between simple and more complex references to children’s mental states (e.g., “*She remembers what she used to be able to do and that makes her more upset with her current difficulties*”). One useful avenue for future research would therefore be to examine whether the complexity as well as the valence of mind-mindedness is of value in predicting child outcomes.

A second direction would be to explore the extent to which mind-mindedness provides a valuable foundation for parental autonomy support. During the home visits conducted for the current study, a subsample of 87 families was filmed in a variety of parent-child interactions. By examining mother-child discussions of topics of conflict and coding key features (e.g., conflict resolution strategies) we hope in our future work to identify the mechanisms mediating the association between mind-mindedness and children’s disruptive behavior.

### Clinical Implications

In their review of parental and child cognitions, Bugental and Johnston (2000) noted an emerging interest in clinical applications of research findings in order to remediate and prevent family problems. In this regard, one implication of the current set of findings concerns the potential value of applying parental mind-mindedness as a focus for family-based interventions to reduce disruptive behavior in adolescence. For example, mothers suffering from depression have been reported to display low mind-mindedness (Pawlby et al. 2010) and so this focus on mind-mindedness may be particularly helpful for interventions designed to support parents with depressive symptoms. Support for this view comes from recent work demonstrating the effectiveness of video-guided feedback to enhance mind-mindedness in parents with depression (Schacht et al. 2015). It is also worth noting that, even in the context of family adversity, a sizeable proportion of our study mothers were able to tune into their children’s thoughts and feelings. This finding is, in and of itself, encouraging for health professionals interested in developing interventions to promote maternal mind-mindedness. Finally, our interaction findings suggest that such interventions are likely to produce strongest benefits among pre-adolescents most at risk of developing disruptive behavior. Our hope is that the findings from the current study will stimulate others to take up this challenge in order to develop interventions, both to translate research findings into effective strategies for improving child outcomes and to test the diverse theories regarding potential mechanisms underpinning the association between parental cognitions and children’s behavior.

## References

[CR1] Ainsworth M, Bell S, Stayton D, Richards M (1974). Infant-mother attachment and social development. The introduction of the child into a social world.

[CR2] Alemany S, Rijsdijk F, Haworth C, Fañanás L, Plomin R (2013). Genetic origin of the relationship between parental negativity and behavior problems from early childhood to adolescence: a longitudinal genetically sensitive study. Development and Psychopathology.

[CR3] Apperly I, Samson D, Humphreys G (2009). Studies of adults can inform accounts of theory of mind development. Developmental Psychology.

[CR4] Aunola K, Nurmi J (2005). The role of parenting styles in children’s problem behavior. Child Development.

[CR5] Barreto AL, Fearon P, Osório A, Meins E, Martins C (2015). Are adult mentalizing abilities associated with mind-mindedness?. International Journal of Behavioral Development.

[CR6] Beck A, Ward C, Mendelson M, Mock J, Erbaugh J (1961). An inventory for measuring depression. Archives of General Psychiatry.

[CR7] Belsky J (1984). The determinants of parenting: a process model. Child Development.

[CR8] Benbassat N, Priel B (2012). Parenting and adolescent adjustment: the role of parental reflective function. Journal of Adolescence.

[CR9] Bernier A, Carlson S, Whipple N (2010). From external regulation to self-regulation: early parenting precursors of young children’s executive functioning. Child Development.

[CR10] Bhavnagri N, Parke R (1991). Parents as direct facilitators of children’s peer relationships: effects of age of child and sex of parent. Journal of Social and Personal Relationships.

[CR11] Bögels S, Brechman-Toussaint M (2006). Family issues in child anxiety: attachment, family functioning, parental rearing and beliefs. Clinical Psychology Review.

[CR12] Bordeleau S, Bernier A, Carrier J (2012). Longitudinal associations between the quality of parent-child interactions and children’s sleep at preschool age. Journal of Family Psychology.

[CR13] Brophy-Herb H, Bocknek E, Vallotton C, Stansbury K, Senehi N, Dalimonte-Merckling D, Lee Y-E (2015). Toddlers with early behavioral problems at higher family demographic risk benefit the most from maternal emotion talk. Journal of Developmental and Behavioral Pediatrics.

[CR14] Brown T (2006). Confirmatory factor analysis for applied research.

[CR15] Bugental D, Johnston C (2000). Parental and child cognitions in the context of the family. Annual Review of Psychology.

[CR16] Currie C, Molcho M, Boyce W, Holstein B, Torsheim T, Richter M (2008). Researching health inequalities in adolescents: the development of the health behavior in school-aged children (HBSC) family affluence scale. Social Science & Medicine.

[CR17] Darling N, Steinberg L (1993). Parenting style as context: an integrative model. Psychological Bulletin.

[CR18] Demers I, Bernier A, Tarabulsy G, Provost M (2010). Maternal and child characteristics as antecedents of maternal mind-mindedness. Infant Mental Health Journal.

[CR19] Dishion T, McMahon R (1998). Parental monitoring and the prevention of child and adolescent problem behavior: a conceptual and empirical formulation. Clinical Child and Family Psychology Review.

[CR20] Ensor R, Roman G, Hart M, Hughes C (2012). Mothers’ depressive symptoms and low mother-toddler mutuality both predict Children’s maladjustment. Infant and Child Development. Special Issue on Families and Psychopathology.

[CR21] Ensor R, Devine R, Marks A, Hughes C (2013). Mothers’ cognitive references to 2-year-olds predict theory of mind at ages 6 and 10. Child Development.

[CR22] Erikson E (1968). *Identity*: *Youth and crisis*.

[CR23] Goodman R (1997). The strengths and difficulties questionnaire: a research note. Journal of Child Psychology and Psychiatry.

[CR24] Hart D, Fegley S (1995). Prosocial behavior and caring in adolescence: relations to self-understanding and social judgment. Child Development.

[CR25] Harter S (1982). The perceived competence scale for children. Child Development.

[CR26] Hoeve M, Dubas J, Eichelsheim V, Van Der Laan P, Smeenk W, Gerris J (2009). The relationship between parenting and delinquency: a meta-analysis. Journal of Abnormal Child Psychology.

[CR27] Holmbeck GN (2002). Post-hoc probing of significant moderational and mediational effects in studies of pediatric populations. Journal of Pediatric Psychology.

[CR28] Hughes C (2011). *Social Understanding*, *Social Lives*: *from toddlerhood through to the transition to school*.

[CR29] Hughes C, Devine R, Lamb M (2014). A social perspective on theory of mind. Handbook of child psychology and developmental science.

[CR30] Hughes C, Ensor R (2005). Theory of mind and executive function in 2-year-olds: a family affair?. Developmental Neuropsychology.

[CR31] Hughes C, White A, Sharpen J, Dunn J (2000). Antisocial, angry and unsympathetic: ‘hard to manage’ preschoolers’ peer problems, and possible social and cognitive influences. Journal of Child Psychology and Psychiatry.

[CR32] Jaffee S, Caspi A, Moffitt T, Polo-Tomas M, Taylor A (2007). Individual, family, and neighborhood factors distinguish resilient from non-resilient maltreated children: a cumulative stressors model. Child Abuse & Neglect.

[CR33] Kochanska G (1990). Maternal beliefs as long-term predictors of mother-child interaction and report. Child Development.

[CR34] Kutner M, Nachtsheim C, Neter J (2004). Applied linear regression models.

[CR35] Loeber R, Hay D (1997). Key issues in the development of aggression and violence from childhood to early adulthood. Annual Review of Psychology.

[CR36] Lundy BL (2013). Paternal and maternal mind-mindedness and Preschoolers’ theory of mind: the mediating role of interactional attunement. Social Development.

[CR37] Magana A, Goldtein M, Karno M, Miklowitz D, Jenkins J, Fallon I (1986). A brief method for assessing expressed emotion in relatives of psychiatric patients. Psychiatry Research.

[CR38] Maughan B, Rowe R, Messer J, Goodman R, Meltzer H (2004). Conduct disorder and oppositional defiant disorder in a national sample: developmental epidemiology. Journal of Child Psychology and Psychiatry.

[CR39] Meins E, Fernyhough C (2015). *Mind-mindedness coding manual*, *Version 2.2*..

[CR40] Meins E, Fernyhough C, Fradley E, Tuckey M (2001). Rethinking maternal sensitivity: Mothers’ comments on infants’ mental processes predict security of attachment at 12-months. Journal of Child Psychology and Psychiatry.

[CR41] Meins E, Fernyhough C, Wainwright R, Clark Carter D, Gupta M, Fradley E, Tuckey M (2003). Pathways to understanding mind: construct validity and predictive validity of maternal mind-mindedness. Child Development.

[CR42] Meins E, Centifanti L, Fernyhough C, Fishburn S (2013). Maternal mind-mindedness and children’s behavioral difficulties: mitigating the impact of low socioeconomic status. Journal of Abnormal Child Psychology.

[CR43] Meins E, Fernyhough C, Harris-Waller J (2014). Is mind-mindedness trait-like or a quality of close relationships? Evidence from descriptions of significant others, famous people, and works of art. Cognition.

[CR44] Morales J, Guerra N (2006). Effects of multiple context and cumulative stress on urban children’s adjustment in elementary school. Child Development.

[CR45] Muthén B, Muthén L (2012). *MPlus*: *Statistical Analysis with Latent Variables*.

[CR46] Pawlby S, Fernyhough C, Meins E, Pariante C, Seneviratne G, Bentall R (2010). Mind-mindedness and maternal responsiveness in infant-mother interactions in mothers with severe mental illness. Psychological Medicine.

[CR47] Reznick J, Zelazo PD, Astington JW, Olson DR (1999). Influences on maternal attribution of infant intentionality. *Developing theories of intention*: *Social understanding and self control*.

[CR48] Rigby K, Slee P (1993). Dimensions of interpersonal relation among Australian children and implications for psychological well-being. The Journal of Social Psychology.

[CR49] Ryan R, Deci E, Grolnick W, Cicchetti D, Cohen D (1995). Autonomy, relatedness, and the self: their relation to development and psychopathology. *Developmental psychopathology*: *Theory and methods*.

[CR50] Schacht R, Meins E, Fernyhough C, Pawlby S (2015). *Mind-Mindedness in Mothers Hospitalised for Severe Mental Illness*. Paper presented at the biennial meeting of the Society for Research in child development.

[CR51] Schiborr J, Lotzin A, Romer G, Schulte-Markwort M, Ramsauer B (2013). Child-focused maternal mentalization: a systematic review of measurement tools from birth to three. Measurement.

[CR52] Sharp C, Venta A, Midgley N, Vrouva I (2013). Mentalizing problems in children and adolescents. *Minding the child*: *Mentalization-based interventions with children*, *young people and their families*.

[CR53] Shelton K, Frick P, Wootton J (1996). Assessment of parenting practices in families of elementary school-age children. Journal of Clinical Child Psychology.

[CR54] Sher-Censor E (2015). Five minute speech sample in developmental research: a review. Developmental Review.

[CR55] Soenens B, Vansteenkiste M, Smits I, Lowet K, Goossens L (2007). The role of intrusive parenting in the relationship between peer management strategies and peer affiliation. Journal of Applied Developmental Psychology.

[CR56] Sunnafrank M, Ramirez A (2004). At first sight: persistent relational effects of get-acquainted conversations. Journal of Social and Personal Relationships.

[CR57] Walker T, Wheatcroft R, Camic P (2012). Mind-mindedness in parents of pre-schoolers: a comparison between clinical and community samples. Clinical Child Psychology and Psychiatry.

